# TLR7 modulating B-cell immune responses in the spleen of C57BL/6 mice infected with *Schistosoma japonicum*

**DOI:** 10.1371/journal.pntd.0009943

**Published:** 2021-11-17

**Authors:** Haixia Wei, Hongyan Xie, Jiale Qu, Anqi Xie, Shihao Xie, He Huang, Jiajie Li, Chao Fang, Feihu Shi, Huaina Qiu, Yanwei Qi, Xu Tian, Quan Yang, Jun Huang

**Affiliations:** 1 Key Laboratory of Immunology, State Key Laboratory of Respiratory Disease, Guangzhou Institute of Respiratory Health, The First Affiliated Hospital of Guangzhou Medical University, Guangzhou, China; 2 Key Laboratory of Molecular Target & Clinical Pharmacology and the State Key Laboratory of Respiratory Disease, School of Pharmaceutical Sciences & The Fifth Affiliated Hospital, Guangzhou Medical University, Guangzhou, China; 3 China Sino-French Hoffmann Institute, Guangzhou Medical University, Guangzhou, China; University of Passo Fundo: Universidade de Passo Fundo, BRAZIL

## Abstract

B cells played an important role in *Schistosoma* infection-induced diseases. TLR7 is an intracellular member of the innate immune receptor. The role of TLR7 on B cells mediated immune response is still unclear. Here, C57BL/6 mice were percutaneously infected by *S*. *japonicum* for 5–6 weeks. The percentages and numbers of B cells increased in the infected mice (*p* < 0.05), and many activation and function associated molecules were also changed on B cells. More splenic cells of the infected mice expressed TLR7, and B cells were served as the main cell population. Moreover, a lower level of soluble egg antigen (SEA) specific antibody and less activation associated molecules were found on the surface of splenic B cells from *S*. *japonicum* infected TLR7 gene knockout (TLR7 KO) mice compared to infected wild type (WT) mice (*p* < 0.05). Additionally, SEA showed a little higher ability in inducing the activation of B cells from naive WT mice than TLR7 KO mice (*p* < 0.05). Finally, the effects of TLR7 on B cells are dependent on the activation of NF-κB p65. Altogether, TLR7 was found modulating the splenic B cell responses in *S*. *japonicum* infected C57BL/6 mice.

## Author summary

Schistosomiasis seriously jeopardizes public health and social development in tropical and subtropical regions. B cells play an important role in *Schistosoma* infection-induced diseases. TLR7 is an intracellular member of the innate immune receptor. The role of TLR7 on B cells mediated immune response is still unclear. Here, we found the percentage and numbers of B cells increased in the infected mice (*p* < 0.05), and the expression of many activation and function associated molecules were also changed on B cells. B cells were served as the main cell population expressed TLR7. When TLR7 gene was knockout, the soluble egg antigen (SEA) specific antibody and activation associated molecules were decreased in *S*. *japonicum* infected mice. Additionally, SEA could induce the activation of B cells by TLR7. Finally, we found the effects of TLR7 on B cells are dependent on the activation of NF-κB p65. In this study, the characteristic of B cells in the spleen of *S*. *japonicum* infected C57BL/6 mice was explored, and the role of TLR7 on the progress of B cell activation and differentiation was investigated.

## Introduction

Schistosomiasis is a zoonotic parasitic disease that is prevalent in the world, which seriously jeopardizes public health and social development in tropical and subtropical regions [1]. Eggs laid out by the infective parasite mainly deposited in the livers of infected animals and humans, and induced liver granuloma and fibrosis [2,3]. A large number of studies have demonstrated that the soluble egg antigen (SEA) are considered to be the most important antigens to induce the development of Th2 responses during schistosomiasis, and are also the initiating factors for the formation of liver granuloma [4,5]. Additionally, soluble worm antigen (SWA) is another antigen that could induce a liquid immune response in *S*. *japonicum* infected mice [6].

The spleen is the largest peripheral lymphoid organ in the body, which contains a large number of immune cells, including T cells, B cells, and macrophages, and so on. It is well known that splenomegaly is a characteristic symptom of schistosomiasis [7]. Sometimes, egg granulomas will form in the spleens of mice with late *S*. *japonicum* infection which alters splenic morphology [8].

B cells are divided into B1 cells and B2 cells. B1 cells are the main source of natural IgM and provided the first line of defense against infection [9], which account for approximately 5% to 10% of the total number of B cells, and can be subdivided into B1a (CD19^+^ CD11b^+^ CD5^+^) cells and B1b (CD19^+^ CD11b^+^ CD5^-^) Cells [10]. B2 cells mainly settle in the spleen and lymph nodes which proliferate and differentiate after receiving antigenic stimulation. During differentiation, B cells develop an immune response outside the lymphoid tissue follicles, and some of them continue to proliferate and differentiate into plasma cells (B220^-^ CD138^+^) that produce low-affinity antibodies (mainly IgM); another part of the proliferating B cells differentiate into germinal center B lymphocytes (GC B) with a surface marker CD19^+^ CD95^+^ GL7^+^ [11]. Most GC B cells also differentiate into plasma cells and produce antibodies that exert immune functions [12]. A small number of B2 cells differentiate into memory B cells (CD19^+^ CD27^+^), rapidly and strongly differentiate, and proliferate in response to antigen-stimulated again [13]. Memory B cells are divided into two categories: post-switch B memory cells (CD19^+^ CD27^+^ IgD^-^) and pre-switch B memory cell (CD19^+^ CD27^+^ IgD^+^) [14,15].

As important immune cells, B cells located in the center of humoral immunity which could proliferate and differentiate into plasma cells, synthesizing and secreting specific antibodies [14]. In addition to secreting antibodies, B cells can also serve as professional antigen presenting cells (APCs), taking up, processing, treating antigens, and expressing antigen information on the membrane surface in the form of antigen peptide/MHC molecule complexes [16]. The most prominent feature of B cells, compared to other APCs, is the uptake of soluble antigens [17]. The antigen presenting function of B cells plays a key role in the immune response induced by TD antigen, and plays a leading role in the re-immune response. Since B cells taking in antigens through the specific recognition and binding of cell surface BCR, the efficiency is high. Very low levels of the soluble antigen can be presented to T cells [18]. Studies have shown that antigen-specific B cells have an efficient presentation of extremely low concentrations of antigens, which are estimated to be10^2^-10^5^ times lower than the concentration of antigens required by other professional APCs [19].

Toll-like receptors (TLRs) are pattern-recognition receptors that recognize pathogen-associated molecular patterns (PAMPs) and damage-associated molecular patterns [20]. Many kinds of TLRs were found expressed on B cells which can specifically recognize PAMPs on the surface of pathogens to initiate immediate effects, and ultimately determine the direction of acquired immune effects [21,22]. TLRs mediating B cell response was reported to play obvious roles in systemic lupus erythematosus [23], mantle cell lymphoma [24], and inflammation [25]. It has been reported that TLRs and B lymphocyte stimulators may combine to form amplification signals to promote the production of specific antibodies [26].

TLR7 is an intracellular member of the innate immune receptor that recognizes intracellular single-stranded and double-stranded RNA [27]. TLR7 is expressed in many kinds of innate immune cells, such as dendritic cells (DC), monocytes, and macrophages [28–30]. TLR7 agonist could enhance the anti-tumor efficacy of obinutuzumab in murine lymphoma models via NK cells and CD4^+^ T cells [31].

In this study, the characteristics of B cells in the spleen of *S*. *japonicum* infected C57BL/6 mice were explored, and the role of TLR7 on the progress of B cell activation and differentiation was investigated.

## Result

### Splenic B cells content and subset changes in *S*. *japonicum* infected mice

**Fig 1:**
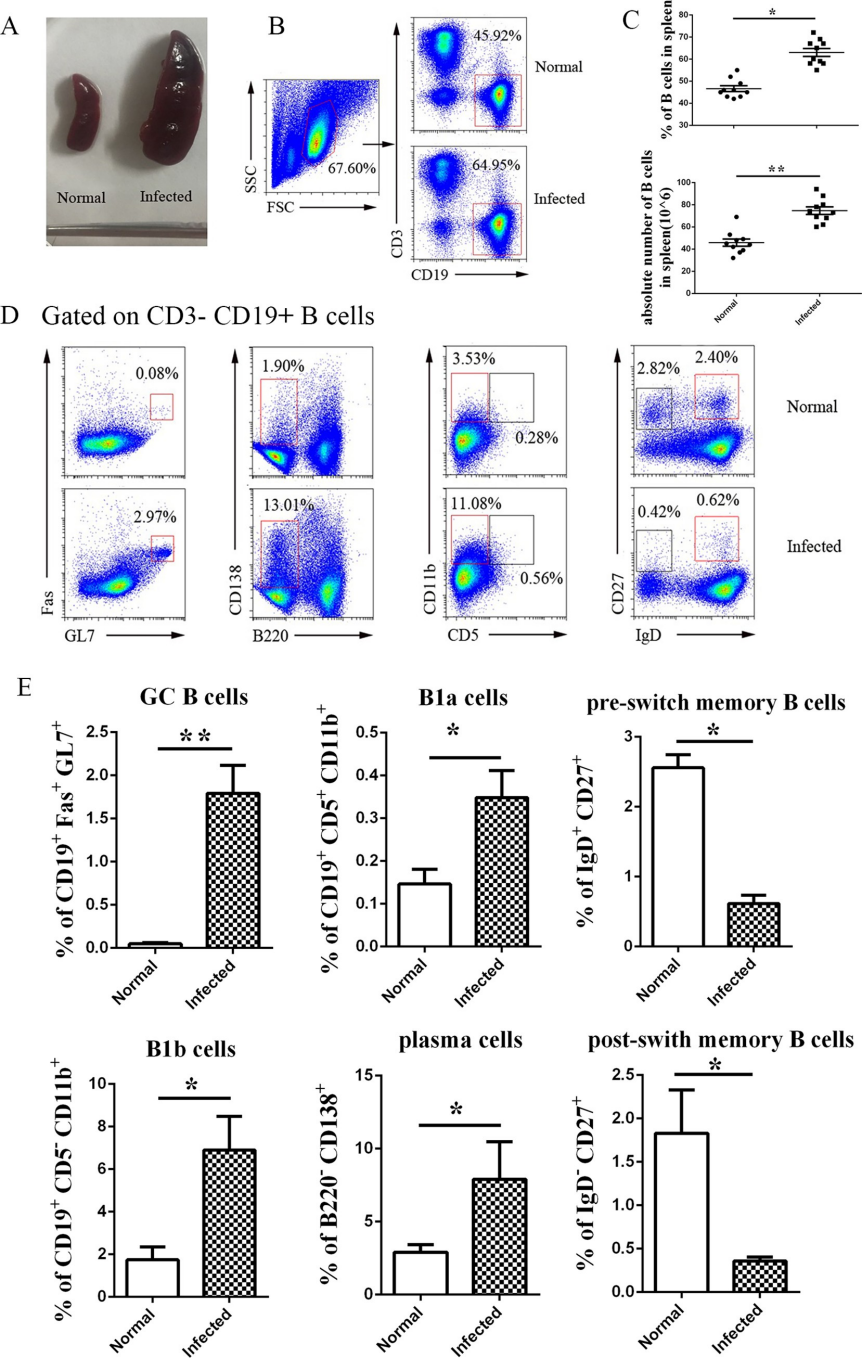
Content and sub-population changes of splenic B cells from *S*. *japonicum* infected mice. C57BL/6 mice were infected by *S*. *japonicum*. 5–6 weeks later, the spleens were picked out, and pictured. Changes in the appearance of spleens from normal and infected mice were shown (A). (B-C) Single cell population of spleen was prepared, the content of CD19^+^ B cells was detected by FCM, the percentage and absolute number (C) of CD19^+^ B lymphocytes in spleens of both normal and infected mice were shown. (D-E) The percentages of different subsets in B lymphocytes from normal and infected mice were detected by FCM. Statistical analysis from three independent experiments was shown. At least 3 mice were used in each group for every independent experiment. ** *p <* 0.01, * *p <* 0.05. Error bar, mean with SEM.

To detect the change of splenic B cell in *S*. *japonicum* infected mice, C57BL/6 mice were infected with schistosome cercariae. 6 weeks after infection, the spleen was picked out and snapped. As showed in Fig 1A, the mouse spleen was significantly enlarged after *S*. *japonicum* infection. It suggested a huge immune response was induced in the bodies of the infected mice as reported [7]. Then the single nuclear cell solution was prepared, and stained by different fluorescein labeled anti-CD3 and anti-CD19 mAb (Fig 1B). Flow cytometry (FCM) results showed that the percentage and the absolute number of splenic B cells in the infected mice increased significantly (Fig 1C, *p* < 0.05). It demonstrated that splenic B cells were involved in the course of *S*. *japonicum* infection. To further identify the sub-population of B cells which play more effects in the course of *S*. *japonicum* infection, the expression of Fas, GL7, CD138, B220, CD5, CD11b, IgD, and CD27 on CD19^+^ B cells were detected by the means of cell surface staining (Fig 1D). Results (Fig 1E) showed that in CD19^+^ splenic B cell population, the percentage of Fas^+^GL7^+^ germinal center B cell, CD138^+^ effect B cells, CD11b^+^CD5^+^ B1a cells, and the CD19^+^CD11b^+^CD5^-^ B1b cells were significantly higher in the infected mice than those in the normal control mice (*p* < 0.05). However, the percentages of CD27^+^ memory B cells populations in infected mice were significantly lower than that in the normal mice (*p* < 0.05). It demonstrated that *S*. *japonicum* could induce innate and adaptive B cells response at 6–8 weeks post infection.

### Functional changes of splenic B cells in *S*. *japonicum* infected mice

**Fig 2:**
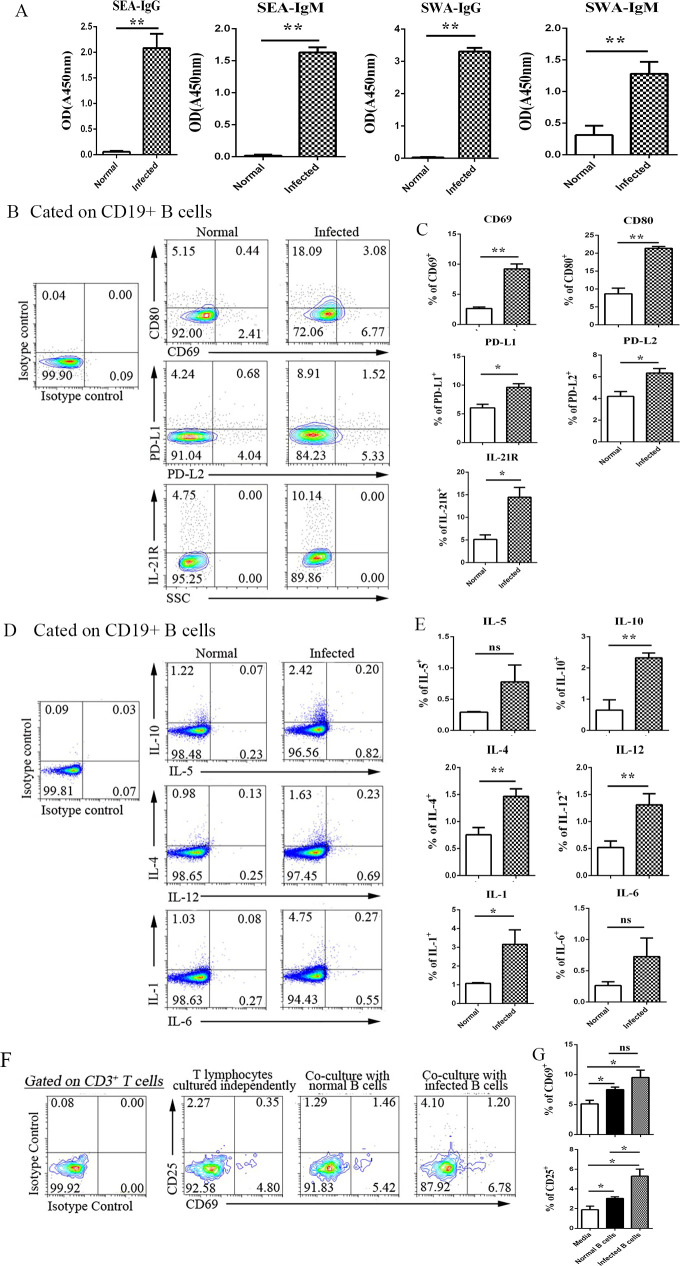
Activation and function of splenic B lymphocytes during infection of *S*. *japonicum*. C57BL/6 mice were infected by *S*. *japonicum*. 5–6 weeks later, blood was collected, specific antibodies in serum against SEA and SWA were detected by ELISA (A). (B-C) Spleen was picked out, single cell population of spleen was prepared. The expression of CD69, CD80, PD-L1, and PD-L2 on splenic B cells was detected by FCM. (B) One typical result. (C) Statistical analysis of the proportions of CD69, CD80, PD-L1, and PD-L2 expressing B lymphocytes. (D-E) Splenocytes were stimulated by PMA plus ionomycin for 4.5 h, the percentages of IL-1, IL-4, IL-5, IL-6, IL-10, and IL-12 secreting cells were detected by the means of intracellular cytokines staining. (D) One typical result. (E) Statistical analysis of the percentages of different cytokines secreting B cells. (F-G) CD3^+^ T lymphocytes from normal mice sorted by FCM were cultured with CD19^+^ B lymphocytes sorted from normal or infected mice for 3 days. The percentages of CD25 and CD69 expressing T lymphocytes were detected by FCM. One typical result (F) and statistical analysis (G) were shown. Three independent experiments were performed. At least 3 mice were used in each group for every independent experiment. ** *p* < 0.01, * *p* < 0.05, ns *p* > 0.05. Error bar, mean with SEM.

SEA and SWA are major antigens to induce liquid immune response in *S*. *japonicum* infected mice [6]. To explore the roles of B cells in the spleens of *S*. *japonicum* infected mice, the levels of SEA and SWA specific IgG and IgM in the serum of infected mice were detected by ELISA, firstly. As shown in Fig 2A, levels of IgG and IgM in the serum of infected mice increased significantly (*p* < 0.01). It suggested effective B cell response has happened in this model.

In addition to secreting antibodies, B cells can also serve as APCs [16]. To explore the antigen presenting function of B cells, splenocytes were isolated from both normal and *S*. *japonicum* infected mice. The expression of activation and co-stimulation related molecules (CD69, CD80, PD-L1, PD-L2, and IL-21R) were detected on the surface of CD19^+^ B cells. As shown in Fig 2B and 2C, more splenic B cells from *S*. *japonicum* infected mice expressed CD69, CD80, PD-L1, PD-L2, and IL-21R (*p* < 0.05). These suggested that *S*. *japonicum* infection could induce B cell activation, and the active B cells might help T cell activation by expressing co-stimulatory molecules.

Cytokine secreting is another manner of APCs in regulating immune response. Schistosome eggs are important sources of antigen exposed in the host during infection with *S*. *japonicum* and can induce production of Th1, Th2, Th17 and Treg cells and their corresponding cytokines [32]. In this study, the percentages of IL-1, IL-4, IL-5, IL-6, IL-10, and IL-12 secreting B cells were detected by the means of intracellular cytokines staining after stimulated by phorbol 12-myristate 13-acetate (PMA) plus Ionomycin as described in materials and methods. As showed in Fig 2D and 2E, compared to normal group, the percentages of IL-1, IL-4, IL-10, and IL-12 secreting B cells in spleens of infected mice increased significantly (*p* < 0.05), no significant changes were found in the percentages of IL-5 and IL-6 secreting B cells (*p* > 0.05). It suggested that B cells also could mediate the immune response in the spleens of *S*. *japonicum*-infected mice by cytokine secreting.

Moreover, the CD19^+^ B cells from the spleens of normal and *S*. *japonicum*-infected mice were selected by micro-beads, and CD3^+^ T cells from the spleens of normal mice were sorted by FCM. The isolated B and T cells were co-incubated in vitro at a ratio of 1:4 in 24 well plates at 37°C under 5% CO_2_ for 3 days. T cells control was set, and SEA was added to the medium. 3 days later, the cells were collected and washed. The expression of CD25 and CD69 on the surface of CD3^+^ T cells was detected by FCM. As shown in Fig 2F and 2G, the percentages of CD25^+^ cells and CD69^+^ cells in the co-culture group were higher than that in the control T cells (*p* < 0.05). Furtherly, the percentage of CD25^+^ T cells was significantly different between normal and infected B cells co-cultured T cells (*p* < 0.05). These results demonstrated that the *S*. *japonicum* infection inducted B cells could help the activation of T cells in the spleen.

### The expression of TLR7 on B cells in *S*. *japonicum* infection

**Fig 3:**
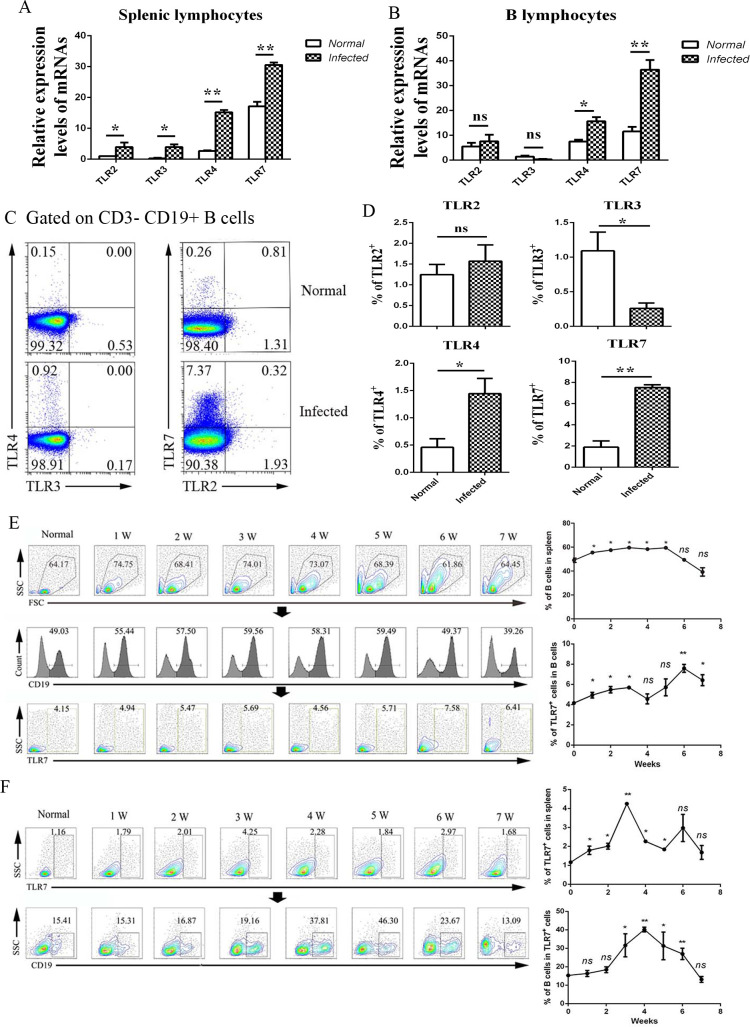
Change of TLRs on B lymphocytes during infection of *S*. *japonicum*. 5–6 weeks post *S*. *japonicum* infection, the spleens were picked out from both normal and infected mice. (A-B) Expression of TLR2, TLR3, TLR4, and TLR7 on lymphocytes (A) or B lymphocytes sorted from spleen (B) was explored by qRT-PCR. (C-D) Expression of TLR2, TLR3, TLR4, and TLR7 on splenic B lymphocytes was detected by FCM. One typical result (C) and statistical analysis (D). (E-F) C57BL/6 mice infected by *S*. *japonicum* were sacrificed weekly from week 1 to week 7, non-infected control was set. The expression of TLR7 on B cells was dynamically detected by FCM. One typical result and statistical analysis was shown. (E) The CD19 expressed B cells were gated from mononuclear cells firstly. The percentages of TLR7 expressing cells from infected mice were compared with the normal group. (F) The TLR7 expressing cells were gated from mononuclear cells firstly. The percentages of CD19 expressed B cells from infected mice were compared with the normal group. Three independent experiments were performed. At least 3 mice were used in each group for every independent experiment. ** *p* < 0.01, * *p* < 0.05, ns p > 0.05. Error bar, mean with SEM.

Many kinds of TLRs were found expressed on B cells during the immune response induced by invaded pathogens [21,22]. To explore the effects of TLRs on B cells in the course of *S*. *japonicum* infection, spleens were picked out from both normal and infected mice. Single nuclear splenocytes were prepared, and B cells were sorted by micro-beads. Total RNA was extracted from both spleen lymphocytes and purified B cell. The expression of TLR2, 3, 4, and 7 were detected by RT-qPCR. As shown in Fig 3A, the expression of TLR2, 3, 4, and 7 in splenocytes increased significantly after infection (*p* < 0.05). In purified B cells, the expression of TLR4 and 7 increased significantly after infection (Fig 3B, *p* < 0.05), especially the expression of TLR7 which increased nearly 3 times after infection. However, no significant change was found in the expression of TLR2 and 3 (*p* > 0.05). It suggested that TLR7 might be the main factor that mediated B cells response in the course of *S*. *japonicum* infection.

Moreover, the expression of TLRs in splenic B cells was examined by FCM. The results (Fig 3C and 3D) showed that the percentages of TLR4 and TLR7 expressing B cells were higher in the spleen of infected mice (*p* < 0.05). The percentage of TLR7^+^ cells (about 7%) is higher than other TLRs in the splenic B cells from infected mice. The percentage of TLR3^+^ B cells slightly decreased (*p* < 0.05) post-infection, and no significant difference was detected in TLR2 expression (*p* > 0.05). These indicated that TLR7 is the main kind of TLRs that could mediated the immune response of B cells in the course of *S*. *japonicum* infection.

Furthermore, the expression of TLR7 on B cells was dynamically detected from week 1 to week 7 after infection. The B cells were gated firstly. As shown in Fig 3E, the percentages of CD19^+^ cells were increased in the population of mononuclear cells after infection and peaked on weeks 3–5. The percentages of TLR7^+^ cells in B lymphocytes also increased after infection. It peaked at about 7.58% on week 6. Then the TLR7 expressing cells were gated. As showed in Fig 3F, the percentages of TLR7^+^ lymphocytes were increased and peaked at about 4.25% on week 3. The percentages of CD19^+^ cells in TLR7 expressed mono-nuclear cells were significantly increased after infection. It sharply increased and peaked on week 4 (about 40%). These results suggested that B cells were the main kind of cells in TLR7 mediated immune response in the course of *S*. *japonicum* infection.

### Roles of TLR7 on *S*. *japonicum* infection induced splenic B cell response

**Fig 4:**
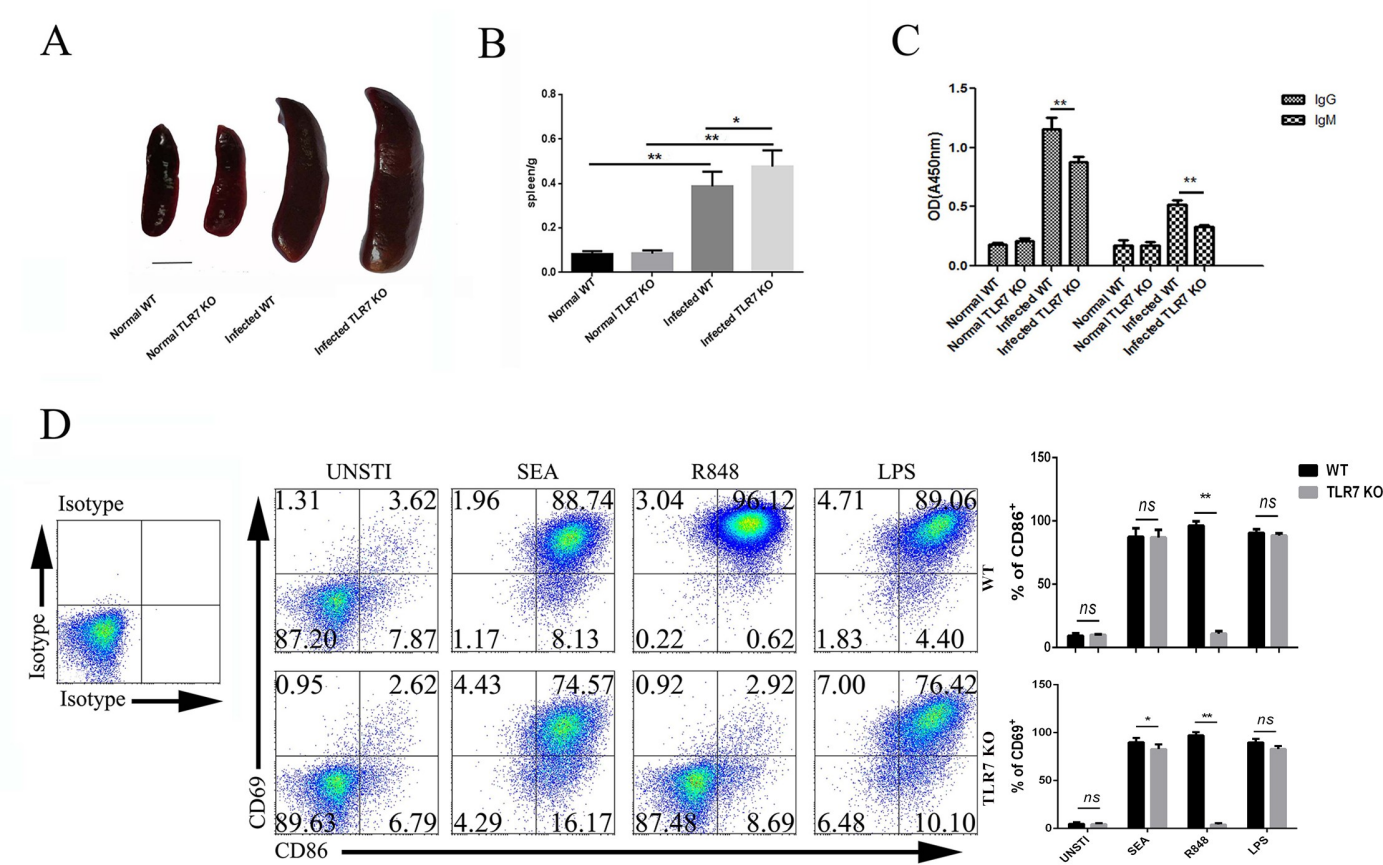
The influence of TLR7 on spleen and splenic B lymphocytes of *S*. *japonicum* infected mice. Both C57BL/6 mice and TLR7 KO mice were infected by *S*. *japonicum*. 5–6 weeks later, spleens were picked out, pictured (A) and weighed (B). bar, 1cm. Blood was collected, specific antibodies in serum against SEA were detected by ELISA (C). (D) B lymphocytes sorted from spleen of normal and TLR7 KO mice were cultured in medium with SEA, R848, or LPS for 3 days, non-stimulated control was set. The expression of CD69 and CD80 on B lymphocytes was detected by FCM. One typical result and statistical analysis was shown. Three independent experiments were performed. At least 3 mice were used in each group for every independent experiment. ** *p* < 0.01, * *p* < 0.05, ns p > 0.05. Error bar, mean with SEM.

To validate the role of TLR7 on *S*. *japonicum* infection induced B cells response, TLR7 knockout (TLR7 KO) mice were infected by *S*. *japonicum*. 5–6 weeks later, the spleens were picked out, pictured, and weighted. As shown in Fig 4A, the *S*. *japonicum* infection induced splenomegaly was increased in the infected TLR7 KO mice. The ratio of the weight of spleen to the weight of isolated mice was also increased in the infected TLR7 KO compared to the infected WT mice (Fig 4B, *p* < 0.05). It suggested TLR7 could inhibit the inflammation response in the spleens of *S*. *japonicum* infected mice.

Moreover, the levels of SEA and SWA specific IgG and IgM in serum of infected TLR7 KO mice were detected by ELISA. Results (Fig 4C) indicated that higher levels of SEA and SWA specific IgG and IgM antibodies were induced in the infected (both WT and TLR7 KO) mice. Compared to the infected WT mice, the levels of both SEA and SWA special IgG and IgM in the infected TLR7 KO mice were lower (*p* < 0.01). These indicated that TLR7 could help antibody producing in *S*. *japonicum* infected mice.

Furthermore, splenic B lymphocytes were sorted from normal WT and TLR7 KO mice, and cultured with or without SEA (100 μg/ml) *in vitro*. 3 days later, cells were collected, and stained by different fluorescence labeled antibodies, the percentages of CD69 and CD80 expressing cells were detected by FCM. Results (Fig 4D) showed more B lymphocytes from both WT and TLR7 KO mice expressed CD69 and CD80 when stimulated with SEA. Compared to B cells from WT mice, the percentages of CD69 expressed B lymphocytes induced by SEA in TLR7 KO mice were lower (*p* < 0.05). These results suggested that B cells could respond directly to SEA, and TLR7 is helpful in SEA induced B cell activation.

**Fig 5:**
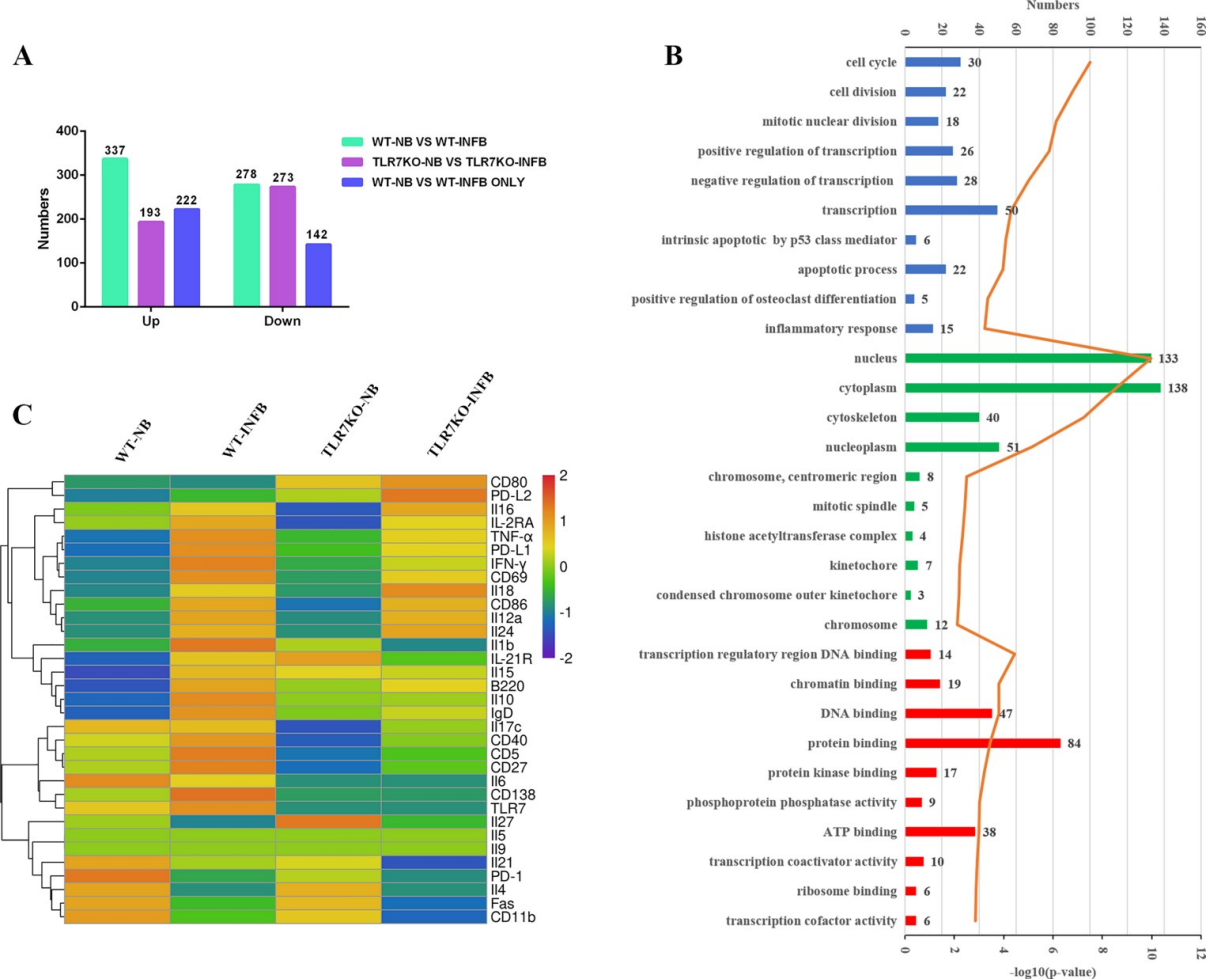
Bioinformatics analysis of the gene expressions of CD19^+^ B cells from the spleens. 5–6 weeks post-infection with *S*. *japonicum*, the B lymphocytes from WT (WT-NB) and TLR7KO(TLR7KO-NB) mice, infected WT (WT-INFB) and TLR7KO(TLR7KO-INFB) mice were isolated, respectively. Then RNAs were extracted immediately and used for RNA sequencing. (A) The numbers of the significantly up-regulated and down-regulated genes in different comparison groups. (B) Gene Ontology (GO) analysis of the 364 ONLY significantly regulated genes in WT-NB and WT-INFB comparison group, which were not significantly regulated in TLR7KO-NB and TLR7KO-INFB comparison group. GO analysis of the 364 genes revealed that they were significantly enriched in the biological processes (blue) of “cell cycle”, “cell division” “mitotic nuclear division”, “transcription regulation”, “apoptotic” and “inflammatory response”. The top 10 terms in cellular components (green) showed they were comprehensively distributed in “nucleus”, “cytoplasm”, “cytoskeleton”, and “nucleoplasm”. The top 10 terms in molecular function (red) indicated that they worked by “binding” to protein, DNA, chromatin, ATP, etc. Stacked bar chart indicated the number of proteins overlapped with the database, and connected orange points represented the logarithm of *p*-values. (C) The expression of some important surface markers and intracellular cytokines from the four groups were compared. PD-L1, CD69, TNF-α, IFN-γ, and IL-10 were up-regulated in the WT-NB and WT-INFB comparison group, while they were almost stable in the TLR7KO -NB and TLR7KO -INFB comparison group.

To gain insight into how does TLR7 regulates B lymphocyte activation, spleens from normal and infected WT and TL7 KO mice were picked out 5–6 weeks after infection, respectively. Single mono-nuclear cells were prepared, and B cells were purified by micro-beads. Then the expression profiles of the B lymphocytes from WT (WT-NB) and infected WT (WT-INFB) mice, TLR7KO (TLR7KO-NB), and TLR7KO (TLR7KO-INFB) mice were sequenced, respectively. The transcriptional signatures differed markedly from each other (S1 Table). The significantly differently expressed genes (fold change ≥ 2, *p* < 0.05) were identified. When compared with WT-NB, there were 337 up-regulated and 278 down-regulated genes in the WT-INFB group, and when compared with TLR7KO-NB, there were 193 up-regulated and 273 down-regulated genes in the TLR7KO-INFB group (Fig 5A and S2 Table).

To further explore the role of TLR7, we just focused on the 222 up-regulated and 142 down-regulated genes which were just significantly changed in the WT-NB and WT-INFB comparison group and were almost stable in the TLR7KO -NB and TLR7KO -INFB comparison group (Fig 5A and S2 Table). To obtain a comprehensive view of the screened genes, GO analysis were performed to identify the significantly enriched functional terms. The results revealed that the 364 screened genes were significantly enriched in a variety of biological processes, cellular components, and molecular functions (*p* < 0.05). As shown in Fig 5B, the top 10 enriched terms within the biological process category were as follows, “cell cycle”, “cell division” “mitotic nuclear division”, “transcription regulation”, “apoptotic” and “inflammatory response” etc. The top 10 terms in cellular components showed they are comprehensively distributed in “nucleus”, “cytoplasm”, “cytoskeleton”, and “nucleoplasm”. The top 10 terms in molecular function indicated that they work by “binding” to protein, DNA, chromatin, ATP, etc. These results show that the genes which were likely regulated by TLR7 may be important for the cell cycle, cell division, cell differentiation and inflammatory response. Additionally, the expression of surface markers and intracellular cytokines from the four groups were compared. As shown in Fig 5C, PD-L1, CD69, TNF-α, IFN-γ, IL-10, were up-regulated in the WT-NB and WT-INFB comparison group, while, they were almost stable in the TLR7 KO-NB and TLR7 KO-INFB comparison group.

**Fig 6:**
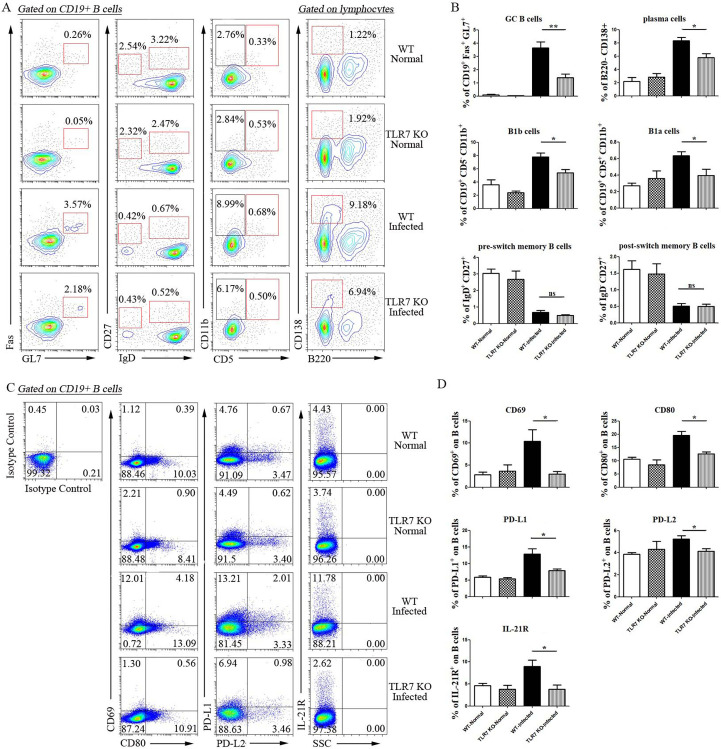
Effect of TLR7 on the subset and phenotype of B lymphocytes during *S*. *japonicum* infection. (A-B) Flow cytometric and statistical analysis of different subsets of B lymphocytes from four groups. A representative analysis(A). (C-D) Expression of CD69, CD80, PD-L1 and PD-L2 on B lymphocytes from four groups was analyzed by the FCM (C) and statistic (D). Statistical analysis from three independent experiments was shown. At least 3 mice were used in each group for every independent experiment. ** *p <* 0.01, * *p <* 0.05, ns *p* > 0.05. Error bar, mean with SEM.

Furtherly, the subsets and activation of B lymphocytes were detected in both normal and *S*. *japonicum* infected WT and TLR7 KO mice. As showed in Fig 6A and 6B, the percentages of germinal center B lymphocytes and regulatory B lymphocytes were decreased obviously in infected TLR7 KO mice compared to infected WT mice (*p* < 0.05). There was no difference in the percentages of memory B cells between the two groups (*p* > 0.05). The percentages of CD69, CD80, PD-L1, PD-L2, and IL-21R expressing B lymphocytes from infected WT mice were higher than those from infected TLR7 KO mice (Fig 6C and 6D, *p* < 0.05). Taken together, these results indicated that TLR7 could regulate B lymphocyte activation, differentiation in the course of *S*. *japonicum* infection.

### The effects of TLR7 on B cells are dependent on the activation of NF-κB p65

**Fig 7:**
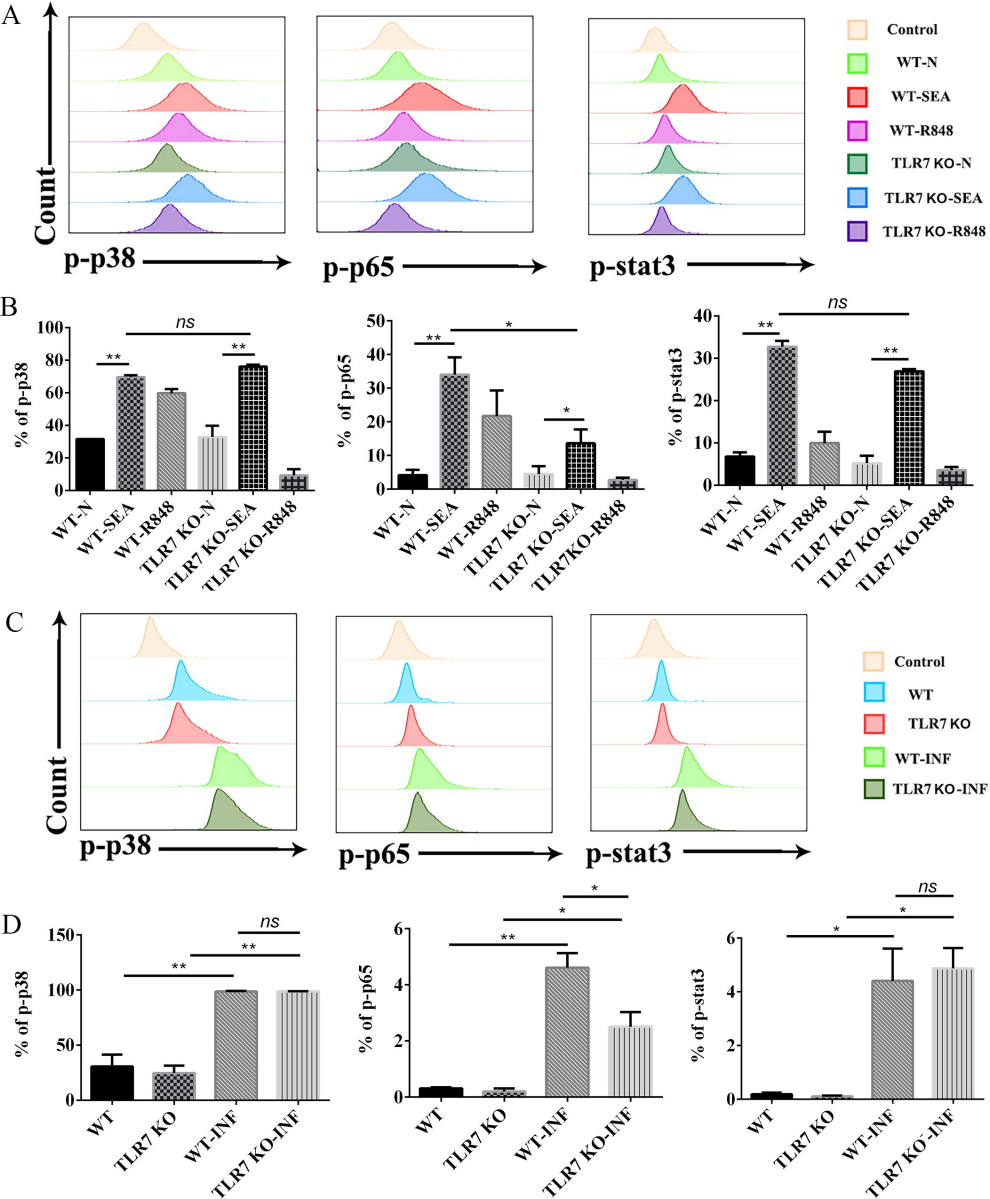
The effects of TLR7 on B cells are dependent on the activation of NF-κB p65. (A-B) Flow cytometric and statistical analysis of the proportions of B lymphocytes with phosphorylated STAT3, p38 and NF-κB p65, and the sorted cells were stimulated with SEA or R848. (C-D) Flow cytometric and statistical analysis of the proportions of splenic B lymphocytes with phosphorylated STAT3, p38 and NF-κB p65 from four groups. Representative analyses (A, C). Statistical analysis from three independent experiments was shown. At least 3 mice were used in each group for every independent experiment. ** *p <* 0.01, * *p <* 0.05, ns *p* > 0.05. Error bar, mean with SEM.

To further explore the mechanism of the effects of TLR7 on B cells, the activation of STAT3, p38 and NF-κB p65 was investigated by FCM. B cells from normal and TLR7 KO mice were sorted, and stimulated with SEA or R848. As shown in Fig 7A and 7B, the proportions of STAT3, p38 and NF-κB p65 phosphorylated B cells in SEA stimulated WT and TLR7 KO cells increased (*p* < 0.05). The proportions of STAT3, p38 phosphorylated B cells from SEA stimulated WT mice cells and TLR7 KO cells were similar (*p* > 0.05), while proportions of p65 phosphorylated B cells from SEA stimulated TLR7 KO cells were decreased compared with that from the WT cells (*p* < 0.05). Similarly, as shown in Fig 7C and 7D, the proportions of STAT3, p38 and NF-κB p65 phosphorylated B cells were increased in the infected mice compared with the uninfected mice (*p* < 0.05). The proportions of STAT3, p38 phosphorylated B cells from WT infected mice were also similar to those from the TLR7 KO infected mice (*p* > 0.05), while proportions of p65 phosphorylated B cells from TLR7 KO infected mice were decreased compared with that from the WT infected mice (*p* < 0.05). These results indicated that TLR7 could regulate B lymphocytes activation, differentiation and cytokine production through TLR7–MyD88–NF-κB pathway in the course of *S*. *japonicum* infection.

## Discussion

Schistosomiasis is a kind of well-known immunologic disease. SEA induced Th2 responses played an important role in the progress of disease development, which could regulate B cells mature, and differentiate into plasma cells [33]. As the spleen is the main B cell living peripheral lymphoid organ, some of the new properties of B cells were investigated in the spleens of *S*. *japonicum* infected mice.

In this manuscript, splenomegaly was found in the infected mice, and significant higher percentage and number of B cells, and different portions of the subsets of B cells were found in the infected mouse spleens. These demonstrated that splenic B cells were involved in the course of *S*. *japonicum* infection. Consistent with us, B cell response is found required for hepatic granuloma formation in the early infection of *S*. *japonicum* [33], and B-cell knockout mice could develop more extensive liver injury following infection with *S*. *mansoni* [34].

Except to act as the antibody secreting cells, B cells are a kind of professional APCs, which could help the activation of antigen specific T cells. In this study, a high level of SEA and SEA specific antibodies, and a higher percentage of splenic CD69^+^ B cells were found in the infected mice (Fig 2A–2C). These indicated the activation of splenic B cells in the progress of *S*. *japonicum* infection. IL-21 was a kind of cytokine mainly secreted by Tfh cells, which could effectively enhance B cell maturity and differentiation [35,36]. Higher level of IL-21R was detected on the surface of infected mouse splenic B cells. It implied that Tfh cells might play an important role in *S*. *japonicum* infection induced splenic B cells activation. CD80 is a typical co-stimulator expressed on the APC, which could bind to CD28 on the T cells, inducing T cell activation. PD-L1 and PD-L2 were mainly expressed on the activated myeloid cells, which could bind to PD-1 on the activated T cells, and induce cytotoxic T cells exhaustion [37]. Higher percentages of CD80, PD-L1, and PD-L2 positive B cells were found in the infected mice (Fig 2C). It suggested B cells could not only helping T cells activation but also inducing cytotoxic T cells functional exhaustion.

Cytokine secreting is another manner of APCs in regulating the immune response. Schistosoma eggs are important sources of antigen exposed in the host during infection with *S*. *japonicum* and can induce the production of Th1, Th2, Th17, and Treg cells and their corresponding cytokines [32]. In this study, the percentages of IL-1, IL-4, IL-10, and IL-12 secreting splenic B cells were increased in the infected mice (Fig 2D and 2E). IL-1 and IL-6 are the central mediators of immunological response secreting by APCs, which show important roles in regulating B and T cell activation [38]. IL-12 is a classic cytokine in inducing Th1 response, which could also drive the differentiation and function of a Tfh1-like cell population [39]. IL-4 is the typic cytokine secreting by Th2 cells, which can promote the occurrence of Th2 response, and is of great significance in inhibiting inflammation. Recently, a group of IL-10 secreting B cells was defined as Breg cells, which can act on related T cells, and exert a negative immunomodulatory function to maintain the body’s immune homeostasis [40]. These demonstrated that *S*. *japonicum* infection-induced splenic B cells could modulate the immune response by secreting cytokines. Consistent with us, B cells were reported to have a significant effect on the systemic anaphylaxis caused by infection with *S*. *mansoni*, and can inhibit inflammation by secreting IL-10 [41]. It is reported that SEA from *S*. *mansoni* can directly stimulate IL-10 secretion from B cells *in vitro* and induce the production of regulatory B cells [42]. Moreover, purified B cells from infected mice could effectively activate co-cultured T cells from naive mice, which further demonstrated that splenic B cells could guide the T cells activation in the course of *S*. *japonicum* infection.

B cells expressing multiple TLRs, which could combine with other stimulators to enforce the function of B cells. This study found that the expression of TLR4 and TLR7 increased obviously after infection, and the percentage of TLR7 expressing B cells was higher than that for TLR4 expressing B cells in the infected mice. Furtherly, B cells were found to be the main kind of cells in TLR7 mediated immune response in the course of *S*. *japonicum* infection (Fig 3). The percentages of B cells in TLR7 expressed cells were significantly increased after infection and peaked at about 40% on week 4 and then decreased rapidly on week 6 and week7. The possible reasons maybe that some B cells were matured and differentiated into plasma cells on week 6–7, which may not express TLR7. The acute infection period for Schistosomiasis is 5 ~ 8 weeks post infection. Various immune cells are activated and may expressed TLR7. For example, our previous study found that the percentage of TLR7 expressed T lymphocytes from the mesenteric lymph node (MLN) of *S*. *japonicum* infected mice increased on week 6 post infection [6].

Moreover, TLR7 KO mice were infected by *S*. *japonicum* to confirm the roles of TLR7 on splenic B cells. Serious splenomegaly was found in infected TLR7 KO mice (Fig 4A and 4B). It suggested that TLR7 could inhibit the inflammation caused by *S*. *japonicum* infection. Consistent with us, TLR7 was reported to orchestrate inflammation and innate immunity upon EV71 infection [43]. On the other hand, ELISA results indicated that the concentration of both SEA and SWA specific IgG and IgM antibodies in serum of the TLR7 KO mice was decreased significantly. These implied that TLR7 played an important role in inducing Th2 response against *S*. *japonicum* infection in both early and later phases. Similarly, TLR7 agonist imiquimod could promote inactivated influenza virus particles induced B cell activation, differentiation, and accelerate antigen specific antibody production [44]. Sorted B cells could respond directly to SEA in our study (Fig 4D). The reason maybe as follows: firstly, our SEA was extracted from the in vitro lysis of Schistosome eggs, which is a crude extract mixture. It is inevitable to have single-stranded and double-stranded RNA inside. In fact, the SEA released from the mature eggs in vivo is also a mixture. Probably, there will be some substances in the mixture, such as exosomes, which contain complex RNA and proteins.

RNA sequencing results showed TLR7 may be involved in the cell cycle, cell division, cell differentiation and inflammatory response (Fig 5). Consistent with it, many activation and association associated molecules were decreased in the splenic B cells from *S*. *japonicum* infected TLR7 KO mice (Fig 6). It further elucidated the role of TLR7 in inducing B cell activation and antigen presentation in the course of S. *japonicum* infection. Similar results have also been reported. TLR7 has been shown to mediate B cell responses to antigen RNA and RNA immune complexes [45,46]. Studies found that activation of the TLR7 pathway could induce differentiation of primary B lymphocytes and antibody class switching, as well as directly affect the expression of cytokines and co-stimulatory molecules by B cells [47]. Alum/TLR7 could induce recruitment of naïve antigen-specific B cells within the draining lymph nodes that might help to sustain the germinal center reaction [48].

TLRs–MyD88–NF-κB p65 was the most classical signaling pathway to stimulate APCs [49,50]. It is also reported that the TLR-MyD88-STAT3 pathway could be activated in human B cells to boost antibody production [51]. *Toxoplasma gondii* dense granule protein GRA24 is reported that could drive MyD88-independent p38 MAPK activation to induce IL-12 production and induction of protective immunity [52]. The activation of p65, STAT3, and p38 were detected (Fig 7) in this study. And the activation of NF-κB p65 was found to be essential for the effects of TLR7 on B lymphocytes activation, differentiation, and cytokine production in the course of *S*. *japonicum* infection.

In summary, TLR7 was found modulating the splenic B cell responses in *Schistosoma japonicum* infected C57BL/6 mice.

## Materials and methods

### Ethics statement

Animal experiments were performed accordance with the regulations and guidelines of the institutional animal care and use committee of Guangzhou Medical University strictly. All protocols for animal use were approved to be appropriate by the institutional animal care and use committee of Guangzhou Medical University (2012–11). Every effort was made to minimize suffering.

### Mice

6–8 weeks old female C57BL/6 mice (Laboratory Animal Centre of Sun Yat-sen University, China), and TLR7 KO mice (B6.129S1-Tlr7^tm1Fl^v/J, the Jackson Laboratory, strains: 008380) were used for the experiments. All mice were maintained in a specific pathogen-free micro-environment at the Laboratory Animal Centre, Guangzhou Medical University.

#### Parasites infection

*The S*. *japonicum* cercariae used in experiments were obtained from *Oncomelania hupensis-*infected snails (Shanghai Institute of Parasitic Disease, China). Wild-type (WT) C57BL/6 mice and TLR7 KO mice were percutaneously infected with 40 ± 5 cercariae, and uninfected WT and TLR7 KO mice were used as control. All mice were sacrificed 5–6 weeks after *S*. *japonicum* infection as previously reported [53].

### Antibodies

The following monoclonal antibodies were used for these studies. APC/cy7 anti-mouse CD3 (145-2C11), Alexa Fluor 647 anti-mouse TLR2 (6C2), PE anti-mouse TLR4 (MTS510), PE anti-mouse TLR7 (A94B10), PE anti-mouse IL-1 (ALF-161), PE anti-mouse IL-4 (11B11), APC anti-mouse IL-5 (TRFK5), PE anti-mouse IL-6 (MP5-20F3), PE anti-mouse IL-10 (JES5-16E3), and PE anti-mouse IL-12 (C15.6) antibodies were purchased from BD Pharmingen (San Diego, CA, USA). PE-cy5 anti-mouse CD19 (6D5), APC anti-mouse B220 (RA3-6B2), PE anti-mouse CD95 (Fas, SA367H8), PE/cy7 anti-mouse CD138 (281–2), FITC anti-mouse CD5 (53–7.3), FITC anti-mouse CD27 (LG.3A10), APC anti-mouse IgD (11-26c.2a), PE anti-mouse CD80 (16-10A1), Brilliant Violet421 anti-mouse PD-L1 (10F.9G2), APC anti-mouse PD-L2 (TY25), PE anti-mouse IL-21R (4A9), APC anti-mouse TLR3 (11F8), FITC anti-mouse GL7 Antigen (T and B cell Activation Marker) (GL7), PE/Cy7 anti-mouse CD11b (M1/70), PE Anti-mouse CD25 (BC96), anti-mouse CD16/32 (Fc Block) (93), PE anti-mouse CD86 (GL-1), and APC anti-mouse CD69 (H1.2F3) antibodies were purchased from BioLegend (San Diego, CA, USA). Phospho-p38 (Thr180/Tyr182) (D3F9) Rabbit mAb, Phospho-Stat3 (Tyr705) (D3A7) Rabbit mAb, Phospho-NF-κB p65 (Ser536) (93H1) Rabbit mAb, and Alexa Fluor 488 Anti-rabbit IgG (H+L) F(ab’)_2_ Fragment antibodies were purchased from CST (Cell Signaling Technology, Inc.).

### SEA and SWA preparation

SEA and SWA were obtained from the Jiangsu Institute of Parasitic Diseases as previously described [54]. In brief, SEA and SWA were sterile filtered and the endotoxin was removed with polymyxin B agarose beads (Sigma-Aldrich). A Limulus amebocyte lysate assay kit (Lonza, Basel, Switzerland) was used to confirm the removal of the endotoxin from SEA and SWA.

### Preparation of splenocytes

Mice were sacrificed after infection for 6 weeks. Spleens were mechanically dissociated and processed through a 100-μm cell strainer (BD Falcon). After erythrocyte was removed by RBC lysis buffer (NH_4_Cl 8.29 g, KHCO_3_ 1 g, Na_2_EDTA, 37.2 mg per liter), the cells were washed twice in Hanks’ balanced salt solution and resuspended in complete RPMI-1640 medium (contained 10% heat-inactivated fetal calf serum, 100 U/ml penicillin, 100 μg/ml streptomycin, 2 mM glutamine, and 50 μM 2-mercaptoethanol).

### Flow cytometry analysis

For cell surface staining, single splenic lymphocyte suspensions were washed twice and stained with anti-CD3, CD19, TLR2, TLR4, B220, CD95 (Fas), CD138, CD5, CD27, IgD, CD69, CD80, PD-L1, PD-L2, GL7, CD11b, CD86, CD25, and IL-21R antibodies for 30 min at 4°C in the dark. Stained cells were washed twice and detected by FCM. For intracellular cytokine staining, single splenic lymphocyte suspensions were incubated for 5 h in the presence of PMA (20 ng/ml, Sigma), ionomycin (1 μg/ml, Sigma) at 37°C under a 5% CO_2_ atmosphere, brefeldin A (10 μg/ml, Sigma) was added 1 h after stimulation. Cells were washed twice and stained with antibodies for cell surface markers for 30 min at 4°C in the dark. Cells were washed again, and fixed with 4% paraformaldehyde, and permeabilized overnight at 4°C in PBS buffers containing 0.1% saponin (Sigma), 1% BSA, and 0.05% NaN_3_. The next day, cells were stained with fluorescence conjugated antibodies specific for IL-1, IL-4, IL-5, IL-6, IL-10, and IL-12 for 30 min. Stained cells were washed twice and detected by using FCM (Cytoflex, Beckman coulter, USA) and data were analyzed by the program CytExpert 1.1 (Beckman coulter, USA). For TLR3, TLR7 detection, cells were fixed and permeabilized without simulated as described as intracellular cytokines staining. For p-p65, p-stat3, p-p38 detection, cells were stained with antibodies of cell surface markers first. They were fixed and permeabilized with transcription factor staining buffer set (Invitrogen, 00-5523-00), and cultured with antibodies specific for p-p65, p-stat3, p-p38. Then the cells were stained with the second antibodies conjugated with fluorescences.

### Cell sorting

Splenocytes were counted under a microscope, and diluted to a final concentration of 2×10^6^ cells/ml for cell culture. CD19^+^ B cells were sorted using mouse CD19 Micro-beads (Miltenyi Biotech, 130-052-201) according to the manufacturer’s instructions. CD3^+^ T cells were stained by FITC labeled anti-mouse CD3 antibody, and sorted by FCM (moflo, Beckman coulter, USA). The purity of T and B cells was above 90%, which were used immediately after sorting.

### RNA preparation for real-time PCR

Total RNA of splenic lymphocytes or B cells was isolated using TRIzol reagent (Invitrogen Life Technologies, Carlsbad, USA), following the manufacturer’s protocol, including DNase treatment of the samples. 1 μg of total RNA was transcribed to cDNA by using a SuperScript III Reverse Transcriptase Kit (Qiagen, Valencia, CA). The following primers were synthesized by Invitrogen (Shanghai, China): for TLR2, 5- AAGATGTCGTTCAAGGAGGTGCG -3 (forward) and 5- ATCCTCTGAGATTTGACGCTTTG -3 (reverse); for TLR3, 5-ATTCGCCCTCCTCTTGAACA -3 (forward) and 5-TCGAGCTGGGTGAGATTTGT-3 (reverse); for TLR4, 5- ACCTGGAATGGGAGGACAATC -3 (forward) and 5-AGGTCCAAGTTGCCGTTTCT-3 (reverse); for TLR7, 5-CCACATTCACTCTCTTCATTGG-3 (forward) and 5-GGTCAAGAACTTCCAGCCTG-3 (reverse); for β-actin, 5-CCGTAAAGACCTCTATGCCAAC-3 (forward) and 5-GGGTGTAAAACGCAGCTCAGTA-3 (reverse). The levels of TLR transcripts were normalized to β-actin transcripts by using the relative quantity (RQ) = 2^-△△Ct^ method.

### ELISA

IgG and IgM antibodies to SEA were measured by ELISA. Briefly, SEA was diluted in coating buffer (0.05 M sodium bicarbonate buffer, pH 9.6) at a concentration of 80 μg/ml (100 μl/well), and the plate was incubated overnight at 4°C. The plate was washed twice, and blocked at 37°C for 1 h by blocking solution (5% skimmed milk powder in 0.02 M PBS with 0.05% Tween-20, pH 7.2). After the wells were emptied, 100 μl of 10-fold diluted serum (1: 100 for IgM; 1:10000 for IgG) was added to each well and incubated at 37° for 1 h. After three times washes, 100 μl horseradish peroxidise (HRP)-conjugated goat anti-mouse IgG (ZB2305; ZSGB-Bio, Beijing, China) and HRP-conjugated goat anti-mouse IgM (RS030210; ImmunoWay Biotechnology, Plano, TX, USA) solution diluted in PBS/Tween-20 was added and incubated at 37° for 1 h. The plate was washed as above, and the reaction was visualized by adding TMB Substrate Reagent (555214, BD), 100 μl per well, for 10 min in the dark at room temperature. The reaction was stopped by stop solution (1 M H_2_SO_4_), 100 μl/well) and the absorbance of each well was measured at 450 nm by ELISA plate reader (Model ELX-800; BioTek).

### RNA sequencing and data analysis

Splenocytes from both normal and infected C57BL/6 or TLR7 KO mice were isolated, respectively. Then, B cells were isolated by CD19^+^ microbeads and RNAs were extracted, respectively. The quality and quantity of RNA were detected by Nanodrop 2000, 260/280 > 2.0 for each sample. The transcriptome sequencing was done and analyzed by the NGS laboratory (No: 80–137533318, GENEWIZ, SZ, China). After sequencing, the raw sequencing reads were filtered. The clean reads were then aligned to the mouse genome. RNA-seq raw sequences deposited in Sequence Read Archive (SRA) are available at NCBI SRA portal with BioProject IDs: PRJNA691716, PRJNA691992, PRJNA691986, PRJNA691995. The mapped reads were assembled and the abundances in fragments per kilobase of exon per million fragments mapped (FPKM) were measured. The differential expressed genes were identified significantly in each comparison with fold change ≥ 2, *p* < 0.05. Bioinformatic analysis was performed using the OmicStudio tools at https://www.omicstudio.cn/tool.

### Statistics

The differences between the two groups were analyzed in Prism (GraphPad Software) using a two-tailed Student’s t-test with equal variance and normal distributions. To compare more than two groups, one-way ANOVA and LSD test by SPSS software package were used with equal variance and normal distributions. Mann-Whitney U test was used with unequal variance or abnormal distributions. The statistical significance was defined as p < 0.05.

## Supporting information

S1 Table The expression profiles of the B lymphocytes from WT (WT-NB) and infected WT (WT-INFB) mice, TLR7KO (TLR7KO-NB) and TLR7KO (TLR7KO-INFB) mice. (XLSX)

S2 Table The significantly differentially expressed genes in each comparison group. (XLSX)
